# Parent-Related Normative Perceptions of Adolescents and Later Weight Control Behavior: Longitudinal Analysis of Cohort Data From Brazil

**DOI:** 10.1016/j.jadohealth.2019.09.007

**Published:** 2020-01

**Authors:** Safa Abdalla, Romina Buffarini, Ann M. Weber, Beniamino Cislaghi, Janaína Calu Costa, Ana Maria B. Menezes, Helen Gonçalves, Fernando C. Wehrmeister, Valerie Meausoone, Cesar G. Victora, Gary L. Darmstadt

**Affiliations:** a Department of Pediatrics, Stanford University School of Medicine, Stanford, California; b Postgraduate Program in Epidemiology, Federal University of Pelotas, Pelotas, Brazil; c Department of Global Health and Development, London School of Hygiene and Tropical Medicine, London, United Kingdom; d Center for Population Health Sciences, Stanford University School of Medicine, Palo Alto, California

**Keywords:** Body image, Body dissatisfaction, Weight management, Dieting, Parental relationships, Parent perceptions

## Abstract

**Purpose:**

Body image–related norms can be imposed by parents and can shape adolescents' body satisfaction in consequential ways, yet evidence on long-term effects is scarce. Longitudinal data from a country with strong body image focus provided a unique opportunity to investigate long-term influences of normative parent-related perceptions.

**Methods:**

Multinomial logistic regression was used on data from a 1993 birth cohort in Brazil to investigate the association of normal–body mass index (BMI) adolescents' perception of their parent's opinion of their weight at age 11 years with their weight control attempts at 18 years, testing a mediating role for body dissatisfaction at age 15 years. All models controlled for body dissatisfaction at age 11 years and BMI change between ages 11 and 15 years.

**Results:**

A total of 1150 boys and 1336 girls were included. Girls were more likely than boys to diet without nutritionist advice to lose weight (51.5% vs. 34.3% among boys) and use medication to gain weight (12.7% vs. 4.2%). Normal-BMI adolescents who reported at age 11 years that their parents thought they were thin had higher odds of feeling thinner than ideal at age 15 years (odds ratio 2.8, 95% confidence interval 1.8–3.2; and odds ratio 2.0, 95% confidence interval 1.5–2.7) among boys and girls, respectively). Feeling thinner than ideal at age 15 years was associated among girls with higher odds of weight gain attempts at age 18 years. Similar patterns appeared among girls reporting that their parents thought they were fat at age 11 years, feeling fatter than ideal at age 15 years and having higher odds of weight loss attempts at age 18 years. Body dissatisfaction was a statistically significant mediator among girls but not boys.

**Conclusions:**

A long-term influence of parent-related perceptions via a likely trajectory of body dissatisfaction is evident among girls.

**Implications and Contribution**

Parents exert an important influence on children's body image. This study revealed a long-term association between what young adolescents believe their parents think about their weight and their attempts to lose or gain weight when they are older. There is need for more attention to understanding influences on adolescent behavior, including the important role of perceived parental opinions.

Normative perceptions or beliefs about what others may consider an ideal body shape and size are a major component of the biopsychosocial model of what causes adolescents' “body dissatisfaction”—negative attitudes and perceptions about one's own body [1]. The model suggests that adolescents' satisfaction with how their body looks is particularly sensitive to sociocultural influences during puberty, a time of important biological and psychological changes. Body satisfaction is profoundly affected by messages conveyed by parents and peers, as well as by the models portrayed in the media [1]. Parents' words and actions (e.g., weight-based teasing, encouragement to control weight, negative weight talk, and dieting) are associated with body dissatisfaction among both boys and girls in different parts of the world [2]. Peers and friends exert their influence through similar mechanisms in addition to making appearance comparisons and judging the appearance of friends [3]. The mass media, including social media, disseminate and glorify body ideals that are not usually attainable for most adolescents [4,5].

These beliefs can be consequential for adolescents' health; body dissatisfaction influences weight control behavior, a common behavior among adolescents [6]. Unhealthy weight control behavior, such as use of diet pills, preludes some eating disorders and may lead to weight gain and poor mental health [6]. These consequences may not be limited to girls; while body dissatisfaction is universally more common among girls, characteristically marked by a desire to be thin, boys are more commonly affected by the drive to appear muscular, documented as early as the age of 8 years [7,8].

The role of parents is particularly important because children as young as preschool age develop attitudes toward their body image that are influenced by their parents' attitudes [9]. At the same time, parents may not be aware of how early their children develop these attitudes [10]. Moreover, the persistence of these parental effects is understudied. The relatively few longitudinal studies in the field had limited follow-up time, long enough (e.g., up to 1 year) to support claims of causal links but in reality, not long enough to explore long-term effects of parent-related influences. On the other hand, a cross-sectional study among women aged 20–35 years reported an association between their body dissatisfaction and the extent to which they remember their parents making comments about their weight when they were younger, suggesting a long-term body dissatisfaction trajectory triggered by parent's weight-related comments [11].

In addition, preventative interventions that involve parents remain inadequately tested [12]; most widely tested interventions tend to work exclusively with adolescents [13,14]. Examining potential long-term influences of adolescents' perceptions of what their parents think about their weight can shed light on the prevention potential of early interventions that are focused on those perceptions and the role of parents in creating them. Therefore, the aim of this work was to investigate the association of perceived parental opinions on body weight in early adolescence with weight control behaviors in late adolescence and gender differences therein in Brazil, a country with a strong focus on body image as evidenced by the second highest number of plastic surgeries and the highest per capita consumption of weight loss medication in the world [[15], [16], [17]].

## Methods

### Design, setting, and data

The present study uses data from the 1993 Pelotas Birth Cohort, a longitudinal study that includes all hospital births during 1993 for women living in Pelotas, a city in southern Brazil. The mothers of 16 newborns declined to participate, and the final cohort included 5,249 children [18,19]. Follow-up visits were restricted to subsamples of the original participants aged 11 years, but the full cohort was visited at ages 11, 15, 18, and 22 years, with follow-up rates of 87.5%, 85.7%, 81.4%, and 76.3%, respectively [20,21]. All visits were carried out by trained interviewers and fieldwork team members. Strategies to locate participants included school census, city census, searches at previous addresses, contacts at known phone numbers, online phonebooks, records from government cash transfer programs, and advertisements in the local media and social networks. At 11 years, the follow-up took place at the participant's house. At 15 years, data were collected at home visits, and several measurements were also taken at the university clinic. At 18 and 22 years, the follow-up took place entirely at the clinic. Further details of the sample and measures have been previously published [[18], [19], [20], [21]]. Questionnaires covered variables related to health (hospitalizations, morbidity, and mental health), schooling, family composition and family characteristics, behaviors and lifestyle (including diet, physical activity, and smoking), discrimination, use of drugs, and violence.

### Conceptual framework

**Figure 1:**
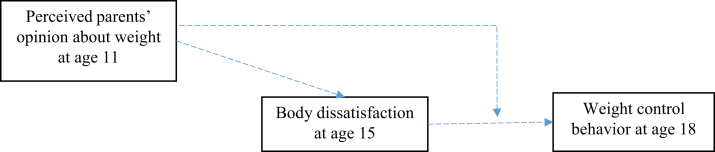
Conceptual framework for the association between perceived parent’s opinion at age 11 y and weight control behavior at age 18 y.

Figure 1 depicts the conceptual framework for this study. Perceived parent's opinion at age 11 years was hypothesized to affect weight control behavior at age 18 years via a trajectory of body dissatisfaction that was captured using dissatisfaction status at age 15 years. Based on finding from the same cohort that perceived parent opinion at age 11 years modified the association between body dissatisfaction at age 15 years and mental health at age 18 years [22], perceived parent opinion in early adolescence was also hypothesized to exert its influence by modifying the association between body dissatisfaction and weight control behavior.

### Inclusions

Only participants who had complete follow-up data from ages 11 years, 15 years, and 18 years for the variables of interest were included. The analysis was also limited to the subset of adolescents who had normal body mass index (BMI) at age 11 years, with the assumption that body dissatisfaction and subsequent weight control behaviors in these individuals would more likely reflect responses of adolescents to what they perceived to be their parents' norms for bodyweight rather than a reflection of true underweight or overweight.

### Description of key variables

#### Perceived parent opinion about weight

Perceived parents' opinion about weight at age 11 years was the measure of normative parent-related perceptions and the main predictor in this study. It was based on the adolescent's response to a question asking them what their parents thought about their weight. The response categories were very fat, fat, normal, thin, or very thin. Because of small sample size in the extreme categories, these options were grouped into three categories: thin and very thin (henceforth called thin), normal, and fat and very fat (henceforth called fat). The variable was considered a measure of the adolescent's awareness of their parent's judgment of their weight based on their parents' own standards for what a normal body weight should be like.

#### Body dissatisfaction

Body image was assessed at ages 11 and 15 years using an age-appropriate figure rating silhouette scale, with nine numbered figures ordered from the smallest to the largest, separate for male and female [23,24]. The adolescents were asked to choose the image that best represented their body (perceived body image) and the figure they would like to be (desired body image). While the numerical difference between the figure numbers is a commonly used and validated measure [25,26], we used a categorical aggregate of the discrepancy for defining body dissatisfaction to clarify the direction of the dissatisfaction and facilitate interpretation of associations with the outcome (weight control behavior). If their perceived body image matched their desired image, the adolescent was considered satisfied with their body. If their perceived body image was smaller than their desired body image, they were considered to feel thinner than ideal. If their perceived body image was larger than their desired body image, they were considered to feel fatter than ideal.

#### Weight control behavior

Weight control behavior at age 18 years was based on adolescents' responses to a question asking if they had done anything to lose or gain weight in the 12 months before the interview. The variable had four response categories: no; yes, to lose weight; yes, to gain weight; and yes, to gain or lose weight. The last category was very small and was excluded (18 adolescents). Those who responded positively to attempting to change their weight were asked to select the methods they used (diet, exercise or sport, medication, tea-like beverage, and others), allowing multiple responses. Responses to a question on whether or not and why the adolescent had seen a nutritionist in the 12 months before the interview were used to identify those who dieted to change their weight with and without guidance from a nutritionist.

#### Body mass index

BMI (weight/height^2^) was calculated using the mean value of two repeated measures of both weight and height. Weight was measured using a digital scale with precision of ±100 g (SECA, Birmingham, UK), and height was measured with a stadiometer with precision of ±1 mm [27]. Interviewers were trained and evaluated before the fieldwork and every 2 months afterward to assess and control intraobserver and interobserver technical measurement errors [18,27]. Normal BMI at age 11 years was defined as having a BMI between −2 and +1 standard deviations from the median BMI for age and sex, according to the World Health Organization standard for children and adolescents [28].

### Analysis

The sex distributions of the variables used in the analyses were described by percentages or means, and the differences between boys and girls were assessed by the chi-square test for categorical variables and *t*-test for difference of means for continuous variables. Multinomial logistic regression was carried out for boys and girls separately with two models: model 1: the association between adolescents' perception of their parents' opinion of their weight at age 11 years and adolescents' body dissatisfaction at age 15 years; and model 2: the association between body dissatisfaction at age 15 years and weight control behavior at age 18 years, adjusting for perceived parents' opinion. Both models were adjusted for baseline body dissatisfaction at age 11 years. Change in BMI between ages 11 and 15 years was also adjusted for as a major biological determinant of the body dissatisfaction trajectory. The role of wealth–total monthly income of all family members in reais—at age 11 years was also tested, given the association found between wealth and body dissatisfaction in a previous birth cohort in the same setting [29]. Interaction of body dissatisfaction at age 15 years with perceived parents' opinion was included in the second model to test for the hypothesized moderating role for parent-related perceptions in the association of body dissatisfaction with weight control behavior. Tested variables with terms that were significant at the .05 level in at least one of the outcome categories were kept in the final models.

The statistical significance of the indirect association between perceived parent opinion and weight change behavior via body dissatisfaction at age 15 years was further tested using the coefficients and their standard errors from the two models, one for the exposure's association with body dissatisfaction at 15 years and the other for the association of body dissatisfaction with weight control behavior. The methodology outlined by Iacobucci was extended to handle the multicategorical exposure, mediator, and outcome variables of the study, testing relative indirect associations (indirect association of one category relative to a reference category) [30,31]. Z-values were computed by dividing each of the two coefficients by its standard error. The two z-values generated were multiplied, and a standard error of the product was calculated. The product was divided by its standard error to produce a z-value for the indirect association, with a two-tailed *p* value. Because eight relative indirect associations (two predictor categories × two mediator categories × two outcome categories) were tested simultaneously, a Bonferroni corrected cut-off *p* value of .00625 was used to determine statistical significance of the indirect association [31].

The analysis was performed with Stata 14 (Stata Corp, College Station, TX).

### Ethical considerations

The Pelotas cohort study was approved by the School of Medicine Ethics Committee of the Federal University of Pelotas. All participants or their legal representatives gave written informed consent before participation in each follow-up. The present secondary analysis was approved by the Stanford Institutional Review Board.

## Results

The analysis included 1150 boys and 1336 girls with normal BMI at age 11 years. Table 1 describes the adolescents' characteristics related to changes in BMI between ages 11 and 15 years, perceived parents' opinion, and body dissatisfaction. Overall, 39.7% of normal BMI adolescents reported that they thought that their parents thought they were thin, whereas only 6.5% reported that they thought that their parents thought they were fat. More boys than girls reported that their parents thought they were thin.

**Table 1:** Characteristics of the 1993 Pelotas birth cohort

	Boys (N= 1150)		Girls (N= 1336)		*p* value^a^
	n	%	n	%	
Perceived parents' opinion at 11 y					.007
Thin/too thin	494	43.0	494	37.0	
Normal	590	51.3	745	55.8	
Fat/too fat	66	5.7	97	7.3	
Body dissatisfaction at 11 y					<.001
Perceive thinner than ideal	357	31.0	355	26.6	
Satisfied	617	53.7	659	49.3	
Perceive fatter than ideal	176	15.3	322	24.1	
Body dissatisfaction at 15 y					<.001
Perceive thinner than ideal	377	32.8	345	25.8	
Satisfied	626	54.48	550	41.2	
Perceive fatter than ideal	147	12.8	441	33.0	
Weight control behavior at 18 y					<.001
No	862	75.0	924	69.2	
Yes, lose weight	99	8.6	270	20.2	
Yes, gain weight	189	16.4	142	10.6	
Methods to lose weight at 18 y					
Diet without nutritionist advice	34	34.3	139	51.5	.003
Medication	7	7.1	11	4.1	.236
Exercise	77	77.8	107	69.3	.108
Tea-like beverages	<5	–	23	8.5	.010
Methods to gain weight at 18 y					
Diet without nutritionist advice	14	7.4	15	10.6	.315
Medication	8	4.2	18	12.7	.005
Exercise	36	19.1	19	13.4	.170
Tea-like beverages	<5	–	<5	–	.839

	Mean (range)	SD	Mean (range)	SD	
BMI (kg/m^2^) at 11 y	16.6 (13.7 to 19.6)	1.3	16.8 (13.3 to 20.7)	1.6	.001
BMI (kg/m^2^) at 15 y	19.5 (14.2 to 28.8)	2.0	20.1 (14.0 to 33.0)	2.3	<.001
BMI change (kg/m^2^) - from 11 to 15 years	2.9 (−2.0 to 11.1)	1.4	3.2 (−1.4 to 13.9)	1.7	<.001

BMI = body mass index; SD = standard deviation.

a Chi-squared test for categorical variables and *t*-test for difference of means for continuous variable.

At age 11 years, girls were more likely to feel fatter than ideal than boys (24.1% vs. 15.3%), and by age 15 years, the difference in prevalence of feeling fatter than ideal had widened between girls (33%) and boys (12.8%). On the other hand, boys were more likely to be satisfied with their body image than girls at ages 11 and 15 years. The mean change in BMI from 11 to 15 years was larger among girls (+3.2 ± 1.7 kg/m^2^) than boys (+2.9 ± 1.4 kg/m^2^).

More girls than boys at age 18 years reported any attempt to lose weight in the 12 months preceding the interview (20.2% vs. 8.6% among boys), whereas boys were more likely to try to gain weight (16.4% vs. 10.6% among girls). Girls were also more likely than boys to diet without a nutritionist advice to lose weight (51.5% vs. 34.3%) and use medication to gain weight (12.7% vs. 7.2%). Although boys were more likely than girls to use exercise to manage their weight, the difference was not statistically significant.

In the multinomial logistic regression models, change in BMI was retained in both models 1 and 2 for boys and girls and wealth in only model 2 for girls. Interaction of perceived parent's opinion with body dissatisfaction at age 15 years was not statistically significant and was therefore not included in model 2.

Among adolescents who had the same body dissatisfaction status at age 11 years, those who reported that their parents thought they were fat at age 11 years had higher odds of feeling fatter than ideal at age 15 years compared with those who reported that their parents thought they were normal (boys: adjusted odds ratio [OR] = 4.3, 95% confidence interval [CI] 2.3–8.1; girls: adjusted OR = 1.8, 95% CI 1.0–3.1; Table 2). Similar findings were observed for adolescents feeling thinner than ideal if they reported that their parents thought they were thin (boys: adjusted OR = 2.4, 95% CI 1.8–3.2; girls: adjusted OR = 2.0, 95% CI 1.5–2.7).

**Table 2:** Association between perceived parent opinion and body dissatisfaction at 15 years by sex, 1993 Pelotas Birth Cohort

	Boys			Girls		
	Adjusted OR	95% CI	*p* value	Adjusted OR	95% CI	*p* value
Outcome category: feeling fatter than ideal versus satisfied						
Perceived parent opinion (ref = normal) at 11						
Thin	0.6	.4–1.0	.037	0.5	.4–.7	<.001
Fat	4.3	2.3–8.1	<.001	1.8	1.0–3.1	.038
Dissatisfaction at 11 (ref = satisfied)			<.001			
Thinner than ideal	1.9	1.2–3.1	.008	1.6	1.1–2.2	.016
Fatter than ideal	3.7	2.3–6.1	<.001	3.9	2.8–5.5	<.001
Change in BMI between ages 11 and 15 y	1.6	1.4–1.8	<.001	1.4	1.3–1.5	<.001
Outcome category: feeling thinner than ideal versus satisfied						
Perceived parent opinion (ref = normal) at 11 y						
Thin	2.4	1.8–3.2	<.001	2.0	1.5–2.7	<.001
Fat	1.2	.6–2.5	.654	1.9	1.0–3.7	.053
Dissatisfaction at 11 y (ref = satisfied)						
Thinner than ideal	2.8	2.1–3.8	<.001	2.6	1.9–3.6	<.001
Fatter than ideal	1.6	1.0–2.4	.038	1.1	.7–1.7	.662
Change in BMI between ages 11 and 15 y	0.8	.7–.9	<.001	0.8	.7–.9	<.001

BMI = body mass index; CI = confidence interval; OR = odds ratio.

At the same baseline body dissatisfaction status at age 11 years (Table 3), feeling fatter than ideal at age 15 years was, in turn, associated among girls with statistically significantly higher odds of trying to lose weight at age 18 years, compared with those who were satisfied (adjusted OR = 1.7, 95% CI 1.3–2.4). Feeling fatter than ideal at age 15 years was also associated with lower odds of trying to gain weight, although the latter association was not statistically significant. Feeling thinner than ideal, again among girls, was significantly associated with lower odds of trying to lose weight (adjusted OR = .6, 95% CI .4–.9) and higher odds of trying to gain weight (adjusted OR = 2.2, 95% CI 1.5–3.3).

**Table 3:** Association of weight control behavior at age 18 y with body dissatisfaction and perceived parent opinion by sex, 1993 Pelotas Birth Cohort

	Boys			Girls		
	Adjusted OR	95%CI	*p* value	Adjusted OR	95%CI	*p* value
Outcome category: trying to lose weight versus no attempt						
Dissatisfaction at 15 y (ref = satisfied)						
Thinner than ideal	0.5	.3–0.9	.026	0.6	.4–.9	.025
Fatter than ideal	1.4	.8–2.5	.249	1.7	1.3–2.4	.001
Dissatisfaction at 11 y (ref = satisfied)						
Thinner than ideal	0.7	.4–1.2	.199	0.8	.5–1.2	.243
Fatter than ideal	1.2	.7–2.1	.487	1.7	1.2–2.4	.002
Perceived parent opinion at 11 y (ref = normal)						
Thin	1.2	.7–1.9	.535	0.7	.5–1.0	.026
Fat	2.7	1.3–5.3	.005	0.9	.5–1.5	.754
Change in BMI between ages 11 and 15 y	1.3	1.1–1.5	.001	1.2	1.1–1.3	<.001
Wealth at 11 y				1.2	1.0–1.3	.005
Outcome category: trying to gain weight versus no attempt						
Dissatisfaction at 15 y (ref = satisfied)						
Thinner than ideal	1.4	1.0–2.0	.064	2.2	1.5–3.3	<.001
Fatter than ideal	0.8	.5–1.6	.582	0.6	.3–1.2	.140
Dissatisfaction at 11 y (ref = satisfied)						
Thinner than ideal	0.8	.6–1.2	.269	1.4	.9–2.1	.155
Fatter than ideal	0.9	.5–1.4	.622	0.4	.2–0.8	.008
Perceived parent opinion at 11 y (ref = normal)						
Thin	1.4	1.0–2.0	.051	1	.7–1.5	.906
Fat	1.0	.4–2.2	.918	0.8	.3–2.1	.722
Change in BMI between ages 11 and 15 y	0.9	.8–1.1	.336	0.8	.7–0.9	.002
Wealth at 11 y				1.1	1.0–1.2	.261

BMI = body mass index; CI = confidence interval; OR = odds ratio.

Among boys, feeling thinner than ideal at age 15 years was associated with lower odds of trying to lose weight (adjusted OR = .5, 95% CI .3–.9) but was not significantly associated with higher odds of trying to gain weight (adjusted OR = 1.4; *p* = .064). However, adolescent boys who reported that their parents thought they were fat had higher odds of trying to lose weight (adjusted OR = 2.7, 95% CI 1.4–5.3). A similarly direct association of reporting that their parents thought they were thin with higher odds of trying to gain weight was marginally statistically significant (adjusted OR = 1.4, *p* = .051).

Further analysis revealed a statistically significant mediatory role for body dissatisfaction at age 15 years among girls (Table 4). Specifically, feeling thinner than ideal was a statistically significant mediator of the association between girls reporting that their parents thought they were thin at age 11 years and attempts to gain weight at age 18 years (*p* = .005).

**Table 4:** Z-value for relative indirect effects of perceived parent's opinion about weight through body dissatisfaction at age 15 y

Perceived parents' opinion	Mediator (body dissatisfaction at age 15 y)	Boys	*p* value	Girls	*p* value
Outcome: trying to lose weight in the 12 mo before age 18 y follow-up					
Thin versus normal	Fatter than ideal versus satisfied	−0.9	.352	−2.6	.009
	Thinner than ideal versus satisfied	−2.1	.040	−2.1	.036
Fat versus normal	Fatter than ideal versus satisfied	1.1	.275	1.7	.085
	Thinner than ideal versus satisfied	−0.4	.688	−1.4	.149
Outcome: trying to gain weight in the 12 mo before age 18 y follow-up					
Thin versus normal	Fatter than ideal versus satisfied	0.5	.629	1.4	.174
	Thinner than ideal versus satisfied	1.7	.081	2.8^a^	.005
Fat versus normal	Fatter than ideal versus satisfied	−0.5	.593	−1.1	.263
	Thinner than ideal versus satisfied	0.4	.700	1.7	.096

a Statistically significant at the Bonferroni corrected α cut-off of .00625.

## Discussion

This study sought to explore possible long-term influences of adolescents' perceptions of their parents' opinion about their weight in a cohort of normal-BMI adolescents from Southern Brazil. The results showed that perceived parent opinion about weight at age 11 years was associated with body dissatisfaction at age 15 years, which in turn, affected weight control behavior in later adolescence. The mediating role of body dissatisfaction as measured in this study was more evident among girls than boys.

The finding that girls were more likely to resort to unhealthy methods to control their weight underscores a detrimental and gendered impact of these perceptions. This mirrors results from other studies in Brazil [17] and is not surprising, given the gendered nature of body norms; boys are usually more likely to want to become lean and muscular, and therefore, they could be more likely to prefer exercise to diet or medication to gain muscle weight or replace fat weight with muscle weight. Moreover, physical activity in Brazil is less common among girls in general, and opportunities for girls to exercise are limited, which could make diet and medication more preferable for girls.

More importantly, the findings imply that parent-related normative perceptions may have long-term consequences. The link of body dissatisfaction at age 15 years with weight control behavior at age 18 years may represent a trajectory of dissatisfaction, lending credence to the connection between exposure at age 11 years and weight control behavior at age 18 years. Although the measure of normative perceptions used in this study does not distinguish between potential causes for those perceptions by adolescents (e.g., parents' positive or negative weight-related comments), studies suggest that such distinction may not be relevant, as even positive comments may emphasize the importance of appearance and were found to be associated with negative outcomes [32]. The results support what was previously suggested by cross-sectional data [11] and further demonstrate a connection between a behavioral outcome and prior body dissatisfaction.

In this study, the perception of the adolescents themselves was considered to be the key factor of interest that drives their behavior, regardless of whether they were correct or not. Although the data for confirming whether the adolescents were accurate in their assessment of their parents' opinions about their weight was not available, the substantial proportion of normal weight adolescents who reported that their parents thought they were thin echoes common reports of parents' tendency to underestimate their children's weight. This is thought to be because of recalibration of visual body norms as a result of rising obesity levels in many parts of the world [[33], [34], [35]]. Brazil is also experiencing a rise in obesity [36], and it is possible that a similar shift in parents' view of children's body norms has taken place. The implications of parents' underestimation of their children's weight for addressing childhood obesity have previously been noted [37]. It is therefore possible that the results represent the impact of mistaking normal weight for thinness, adding a pathway between body image norms and health outcomes that contrasts with that of the classic drive for thinness among girls.

Evidence suggests that the role of parents intersects with that of other sociocultural influences as well as with physical and psychological factors in complex ways. For example, siblings were found to be more likely to engage in weight-based teasing if parents also tease, and the effect of teasing from both parents and siblings on body dissatisfaction was mediated by social comparison (comparison of one's body with that of others) [38]. In addition, parental encouragement to diet and parental negative comments are associated with lower self-esteem [39,40], and self-esteem was shown to protect against body dissatisfaction [41]. The associations found could therefore be a result of those intersections instead of a purely independent effect of parent-related perceptions.

A strength of this study is the use of longitudinal data that spans the adolescent period and that captures a measure of body dissatisfaction midway, providing a plausible, temporally valid link between exposure early in adolescence and outcomes in late adolescence. Some limitations remain, however. The study's nonrandomized design does not account for unmeasured factors. Also, the desire to be muscular, known to be more common among boys, was not measured, and therefore, that dimension of body dissatisfaction was not captured. It is unclear whether that could have affected how boys used the figure rating scale; although the adult figure rating scale was validated in Brazil [42], the adapted age-appropriate version was not, and a study of the validity among men of a commonly used figure rating scale showed that it lacked discriminant validity [43]. More uncertainty is therefore expected around the measure of body dissatisfaction among boys, which may explain the lack of evidence of a mediation role for body dissatisfaction in that group. An additional limitation is that measures of weight control behavior, the measure of how adolescents perceived their parents' opinion about their weight, and the categorical body dissatisfaction measure we used were not subjected to psychometric validation. Finally, lack of suitable measures of peer influence or exposure to body-centered media prohibited exploring how those could intersect with parent-related perceptions.

## Conclusion

Parent-related normative perceptions in early adolescence are associated with weight control behavior in later adolescence, with evidence for a link through a body dissatisfaction trajectory among girls. Pending their replication, these findings imply that considering the role of parents and targeting parent-related normative perceptions may have a larger potential than currently appreciated for limiting the development of unhealthy weight control behaviors later in adolescence and requires further research.
